# Chemical Diversity of Essential Oils from Korean Native Populations of *Agastache rugosa* (Korean Mint)

**DOI:** 10.3390/molecules27196341

**Published:** 2022-09-26

**Authors:** Minji Hong, Ponnuvel Deepa, Ki-Yeon Lee, Kyunghee Kim, Kandhasamy Sowndhararajan, Songmun Kim

**Affiliations:** 1 School of Natural Resources and Environmental Science, Kangwon National University, Chuncheon 24341, Gangwon-do, Korea; 2 Agro-Food Research Institute, Gangwon Agricultural Research & Extension Service, Sinbuk, Chuncheon 24203, Gangwon-do, Korea; 3 Department of Botany, Kongunadu Arts and Science College, Coimbatore 641029, Tamil Nadu, India

**Keywords:** *Agastache rugose*, baechohyang, chemotypes, essential oil, principal component analysis

## Abstract

*Agastache rugosa* (baechohyang) is one of the most important aromatic plants native to the Republic of Korea. *A. rugosa* fragrance has been used to prepare incense since the Goryeo Dynasty in Korea. The present study aimed to explore the variation in the composition of essential oils from *A. rugosa* among native populations in Korea. The seeds of *A. rugosa* were collected from 90 different sites in Korea and seedlings were raised in the nursery. Essential oils were extracted from these populations by the steam distillation extraction method and their chemical compositions were analyzed by GC-MS. The yield of essential oils of *A. rugosa* ranged between 0.11% and 0.86%. A total of 204 components were identified from 90 populations of *A. rugosa*. Out of 204 components, 32 components were common in more than 40 individuals of *A. rugosa* and these 32 components were selected for principal component analysis (PCA). On the basis of the essential oil compositions, six chemotypes—estragole, pulegone, methyl eugenol, menthone, isopulegone, and nepetalactone—were distinguished according to their major components. As a result of the cluster analysis, 90 individuals of *A. rugosa* could be classified into three groups: estragole, methyl eugenol, and pulegone. *A. rugosa* exhibited significant chemical diversity among the individuals. The distribution of chemotypes is associated with the collection of seeds, suggesting that genetic diversity may influence the variations in the chemical compositions and concentrations within the species. This chemical diversity serves as the background to select cultivars for the cultivation and industrial applications of *A. rugosa* cultivars with high essential oil yield and concentration of its chemical components.

## 1. Introduction

*Agastache rugosa* (Fisch. & C. A. Mey.) Kuntze is an important aromatic edible plant in Korea that belongs to the Lamiaceae family. The *A. rugosa* plant is mainly found in East Asia, China, Japan, and Taiwan [1]. The common name of *A. rugosa* is Korean mint (baechohyang) and its aerial parts contain essential oils with a unique fragrance. Therefore, the leaves of *A. rugosa* are used to enhance the aroma and taste of Korean dishes, such as salads and soups [2,3]. In traditional Korean medicine, *A. rugosa* is used for the treatment of numerous diseases, especially anxiety, cholera, diarrhea, fever, and nausea [1,4]. Previously, several studies reported that *A. rugosa* contains a variety of bioactive metabolites that prevent inflammation, cardiovascular diseases, cancer, photoaging, adipogenesis, etc. [4,5,6,7,8]. 

Previously, few studies reported on the chemical composition of essential oils obtained from *A. rugosa* in the Republic of Korea [3,9,10]. This plant has a high developmental value as a flavoring crop for various industrial products. Major biologically active constituents of *A. rugosa* are characterized into two main metabolic classes: terpenoids and phenylpropanoids. The essential oil of *A. rugosa* is mainly composed of estragole, followed by D-limonene, menthone, and pulegone. However, a huge variation in the chemical composition of essential oils of *A. rugosa* has been reported, according to different geographical origins [3,6,11]. Knowledge of the chemical composition of essential oils from aromatic plants is an important quality measure for their commercial utilization. 

Environmental factors play a critical role in determining the chemical polymorphism of plant essential oils [12]. In addition, the quantity and composition of essential oils from aromatic plants are mainly influenced by various factors such as genotype, season, stage of maturity, extraction technique, and drying [13,14]. Principal component analysis (PCA) is an important multivariate analysis method for grouping or clustering quantitative data based on the significance of the sample [11,15,16]. 

In this context, the present study aimed to provide information on the variation of essential oil compositions of Korean *A. rugosa.* For this purpose, seeds of *A. rugosa* were collected from 90 different regions in Korea, the plants were cultivated, and their essential oil compositions were analyzed using gas chromatography and mass spectrometry (GC-MS) analysis. Furthermore, PCA was performed to classify chemotypes in order to identify differences in regional species of *A. rugosa* in Korea.

## 2. Results and Discussion

### 2.1. Color and Yield of Essential Oils from A. rugosa Populations

**Figure 1:**
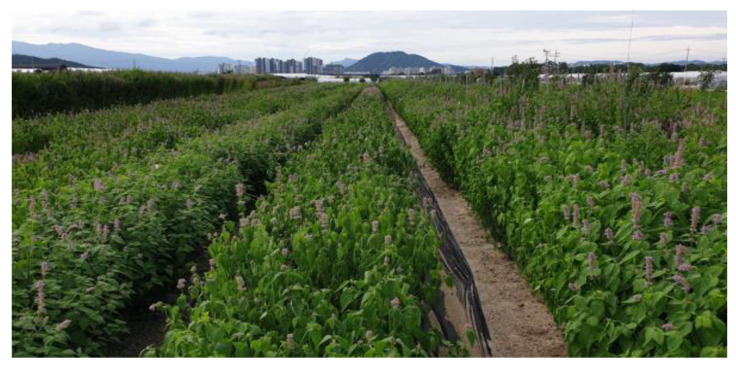
The cultivation of *A. rugosa* under field conditions.

**Figure 2:**
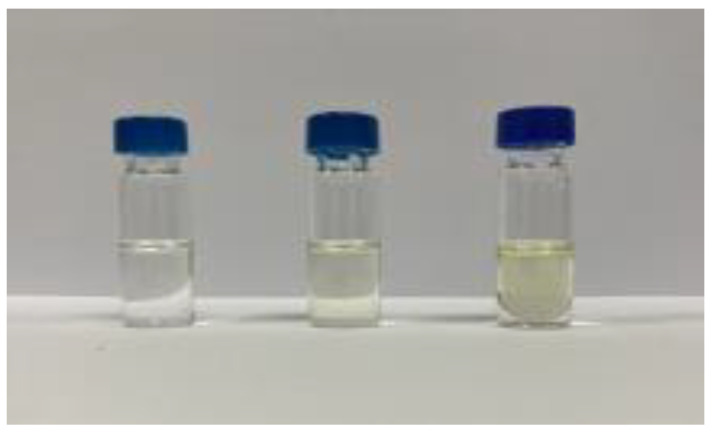
The color of essential oils extracted from *A. rugosa*.

*A. rugosa* is one of the important medicinal and aromatic plants in Korea. The essential oil obtained from this plant has been used for enhancing the aroma of Korean dishes. This oil is mainly composed of monoterpenoids, phenylpropanoids, and other aromatic components [4]. In this study, 90 Korean native *A. rugosa* populations were selected for essential oil analysis. Seeds collected from 90 individuals were cultivated in the same field under similar environmental conditions and then used for essential oil extraction (Figure 1). Essential oils were extracted from 90 individuals of *A. rugosa* by the steam distillation method. For this purpose, seeds of *A. rugosa* were collected from different cultivated regions in Korea. The yield percent (*v*/*w*) and the color of essential oils markedly varied according to the collection sites of the seeds (Table 1). The extraction yield of *A. rugosa* essential oils was a minimum of 0.11% and a maximum of 0.86%. The essential oil varied depending on the seed collection area. Furthermore, the color of the essential oils was classified into colorless, pale lemon, and lemon (Figure 2). Similar to our report, the yield of essential oil obtained from the aerial parts of *A. rugosa* ranged between 0.15 and 0.49% [3,17].

### 2.2. Chemical Composition of Essential Oils of A. rugosa Populations

In total, 204 chemical components were identified in the essential oils of 90 individuals of Korean *A. rugosa* based on the retention indices and mass spectral data (Supplementary Table S1). Among them, methyl chavicol (estragole), D-limonene, isopulegone, menthone, pulegone, β-caryophyllene, and β-cubebene were registered as major components in the essential oils of *A. rugosa* populations. The composition of essential oils of *A. rugosa* populations differed not only by their major components, but also by the number of different classes of components. Li et al. [18] identified 37 chemical components from the essential oil of *A. rugosa*, and the major components were methyl eugenol (50.1%), estragole (8.5%), eugenol (7.5%), thymol (3.6%), and pulegone (2.5%). A recent study reported that the most abundant component in the essential oil of *A. rugosa* was estragole (89.49%), followed by D-limonene (3.40%), menthone (1.80%), and pulegone (1.86%) [3]. Estragole was also the main component in the essential oil of *A. ruogsa* cultivated in Australia [19]. In other studies, menthone (48.8%) was reported as the most abundant component in the essential oil obtained from the leaves of *A. rugosa* cultivated in China [6,20]. Based on previous reports, *A. rugosa* is classified into three chemotypes: pulegone, estragole, and methyl eugenol [18]. These studies suggested that the variation in the essential oil composition may be influenced by various ecological and physiological factors. In addition, ontogenetic cues play a partial role in the varied proportions of essential oil components [11]. 

In the present study, 32 components were common in more than 40 individuals of *A. rugosa. A. rugosa* individuals collected from various sites were classified into six chemotypes according to their chemical components (Table 2). Among them, the estragole chemotype showed the highest number of *A. rugosa* (53 individuals), followed by the pulegone type (17 individuals), methyl eugenol type (11 individuals), menthone type (6 individuals), isopulegone type (2 individuals), and nepetalactone type (1 individual). Although the collected seeds were grown in the same environmental conditions, significant variations were observed in the essential oil compositions of 90 individuals. Hence, the data may reflect the genetic diversity within the species. It was reported that genetic diversity within the species plays a major role in essential oil compositions and concentrations [21,22,23]. Kang et al. [24] studied the genetic diversity of 65 accessions of *A. rugosa* germplasms using inter simple sequence repeat (ISSR) markers. The authors reported these accessions were grouped into two major clusters (A and B), and cluster A is subdivided into two sub-clusters. Furthermore, Dang et al. [25] compared morphological features and essential oil compositions in the pulegone and estragole chemotypes of *A. rugosa.* Previous studies also found that the morphological variations among different chemotypes were mainly in leaf type, trichome density, plant height, and internode length [26,27]. These studies clearly indicate that morphological and genetic variability among *A. rugosa* populations may influence the chemical diversity within species. 

### 2.3. Principal Components Analysis of the Essential Oils of A. rugosa Populations

Multivariate data analysis has been used for the numerical taxonomic classification of plants based on their phenotypic, chemical, and molecular features. In particular, PCA and cluster analyses have been performed on the basis of the essential oil components in plants [11,23,28]. PCA was used to determine the variability in the chemical composition of essential oils obtained from 90 individuals of *A. rugosa* seeds collected from different regions of Korea. Statistical analysis was performed based on the data from the composition of essential oils of *A. rugosa* populations. Based on the principal component analysis of 32 essential oil components from 90 *A. rugosa* individuals, principal components PC1, PC2, PC3, and PC4 accounted for 70.97%, 20.91%, 3.74%, and 3.38% of the total variation, respectively. The main principal components, PC1 and PC2, can account for approximately 91.87% of the total variation. The dimension was reduced to two principal components (Table 3).

**Figure 3:**
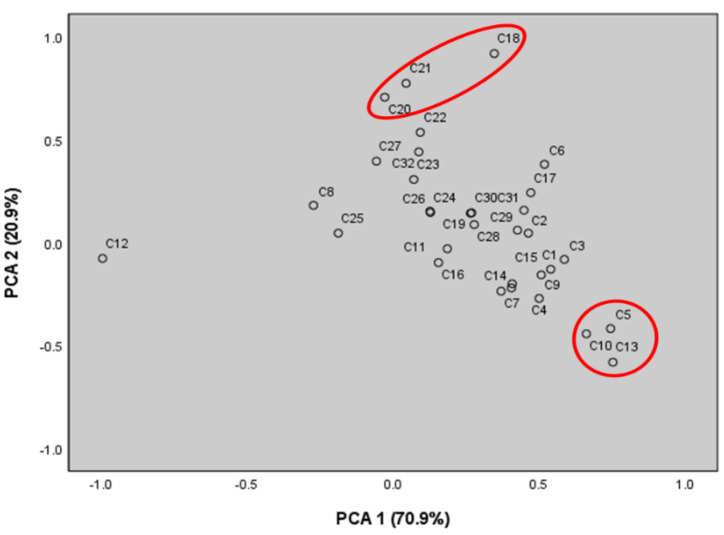
PCA scatter plot for each 32 chemicals from *A. rugosa* essential oils.

Table 4 shows the correlation between chemical components and principal components. The main component PC1 showed a relatively high correlation with the contents of pulegone (C13), β-pinene (C5), and menthone (C10), whereas it showed a low correlation with the content of estragole (C12). On the other hand, the principal component PC2 showed a high correlation with the contents of methyl eugenol (C18), β-caryophyllene (C20), and α-humulene (C21) components. Figure 3 shows the scatter plot of the major essential oil components on the plane coordinates using the principal component PC1 and PC2 values.

**Figure 4:**
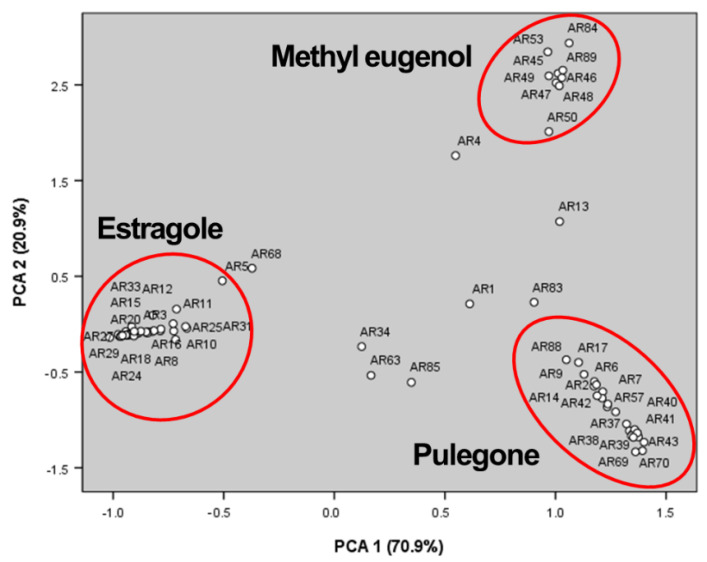
Scatter plot of the 90 *A. rugosa* individuals.

Using the scores of the principal components PC1 and PC2, *A. rugosa* populations have been expressed as a scatter plot, as shown in Figure 4. The principal component PC1 (high content of pulegone, l-β-pinene, and menthone) is correlated with *A. rugosa* individuals of AR2, AR6, AR7, AR9, AR14, AR17, AR21, AR35, AR36, AR37, AR38, AR39, AR40, AR41, AR42, AR43, AR44, AR54, AR57, AR65, AR66, AR69, AR70, and AR88. The principal component PC2 (high content of methyl eugenol, β-caryophyllene, and α-humulene) is correlated with *A. rugosa* individuals of AR45, AR46, AR47, AR48, AR49, AR50, AR53, AR84, and AR89 (Figure 4). 

Zielińska et al. [11] studied the essential oil profiles of the progeny derived from different ages of mother plants of *A. rugosa* in cultivated plants and in vitro shoot cultures. A phenylpropanoid, estragole, was the most abundant component in *A. rugosa* essential oil. The quantitative variation among individuals was mainly in the concentration of estragole, menthone/isomenthone ratio in leaves, and pulegone in inflorescences. The authors suggested that the essential oil compositions of *A. rugosa* were highly dependent on the age of the mother plants. Zielińska and Matkowski [29] reviewed that the essential oil of *A. rugosa* is mainly dominated by estragole, but other monoterpene components such as limonene, menthone, isomenthone, and pulegone may also be present in high concentrations. Similar to our study, Li et al. [18] isolated the essential oils of *A. rugosa* aerial parts collected from Zhejiang, Hubei, and Henan provinces of China and found three different chemotypes, pulegone, estragole, and methyl eugenol, were major components, respectively. The morphological variations, essential oil components, and transcriptomic data between the two chemotypes of *A. rugosa*, pulegone and estragole, were also investigated [25].

The results of the correlation coefficients between 32 chemicals in the essential oils of Korean *A. rugosa* populations are presented in Supplementary Table S2. Among them, the chemical components that showed significance at the 1% probability level and had a correlation coefficient of 0.70 or higher were as follows: 1-octen-3-ol showed a high correlation coefficient with 3-octanone (0.822 **), 3-octanol (0.826 **), and l-β-pinene (0.743 **) components, 3-octanone with 3-octanol (0.795 **), and 3-octanol with β-pinene (0.745 **). β-Pinene showed a correlation with menthone (0.750 **) and pulegone (0.756 **). Isopulegone showed a high positive correlation coefficient with isopiperitenone (0.840 **). β-Bourbonene with β-elemene (0.793 **), methyl eugenol with α-humulene (0.718 **), and β-elemene with β-cubebene (0.711 **). In addition, β-caryophyllene showed the highest correlation coefficients with α-humulene (0.965 **) and β-cubebene (0.729 **). α-Humulene with β-cubebene (0.701 **), and γ-elemene with β-cadinene (0.808**), t-muurolol (0.720 **), and α-cadinol (0.733 **), and β-cadinene showed a positive correlation coefficient with α-cadinol (0.734 **). The biosynthetic pathways of β-caryophyllene and α-humulene are intimately associated because the compounds have the same chemical structures and molecular weights. On the other hand, the strongest negative correlation (−0.716 **) was observed between estragole and β-pinene components. Furthermore, the remaining components showed low correlations. The results demonstrate that the correlations can be used as an important outcome to understand the relationship between Korean native *A. rugosa* populations and their chemical components (Supplementary Table S2). Moreover, the data reveal that cultivars can be developed according to the content of the compounds. The results indicate that *A. rugosa* populations were categorized into three major chemotypes: estragole, pulegone, and methyl eugenol. 

**Figure 5:**
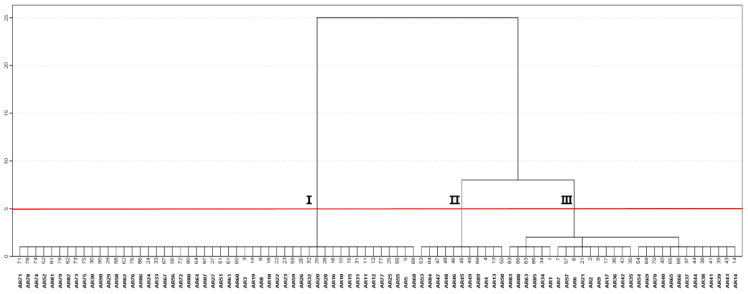
Dendrogram obtained by cluster analysis based on the essential oil components of *A. rugosa* populations.

As a result of the cluster analysis, 90 individuals of *A. rugosa* could be classified into three groups according to their chemical composition, as shown in the dendrogram (Figure 5). Group I included most *A. rugosa* individuals with the highest estragole content in their essential oils. Group II contains individuals of *A. rugosa* with the highest methyl eugenol content and group III contains individuals of *A. rugosa* with the highest pulegone content. Furthermore, group III could be divided into three sub-groups according to the ratio of essential oil components. The chemical composition characteristics of each cluster are shown in Table 5. Dang et al. [25] reported that 46 genes were identified with the biosynthesis of estragole and pulegone. In these, the authors identified chavicol methyl transferase and limonene-3-hydroxylase in *A. rugosa*. Jang et al. [30] investigated the phenotypic expression and floral dimorphism in five Korean populations of *A. rugosa* and reported that three phenotypes were found according to their reproductive characteristics. The findings of this study suggest that different chemotypes among *A. rugosa* populations are not associated with environmental conditions. The changes in the composition of essential oils and the concentration of components may be associated with phenotypic and genetic characteristics of *A. rugosa* populations. In particular, genetic and environmental variations play a key role in phenotypic variation within the species [31].

Previous studies demonstrated that various edaphic and ecological factors influenced the essential oil yield and their chemical components in aromatic plants [23,32,33]. In the present study, the statistical analysis results suggest that the essential oil components are predominantly determined by the plant’s genotype, because all collected seeds were cultivated under similar environmental conditions. Furthermore, planting season may slightly influence the overall yield of essential oils and their compositions of *A. rugosa* populations.

## 3. Materials and Methods

### 3.1. Collection of A. rugosa Seeds and Their Cultivation

Korean domestic *A. rugosa* seeds were collected from 90 different regions of the Republic of Korea in 2019 (Table 1). The seeds were sown in 128-hole seedling trays (17 cm^3^, Seoul Bio Co., Ltd., Chungbuk, Korea) filled with horticultural topsoil in April 2019. The seedlings were grown for 36 days in a glass greenhouse at the Agricultural Research Institute, Gangwon-do Agricultural Research and Development Institute, where the proper temperature (23–25 °C day) and humidity were maintained. In early May, 3.5-leaf-bearing herbaceous seedlings were planted in two rows at 25 cm intervals with an area of 1650 m^2^ (Figure 1). The plants were grown until the flowering stage, which is known to contain the highest essential oil content. After that, aerial parts of all samples of *A. rugosa* were harvested at the blooming stage and immediately cut into 15 cm units used for the essential oil extraction.

### 3.2. Essential Oil Extraction

The essential oils were separately extracted from the aerial parts of 90 different *A. rugosa* individuals by steam distillation. The essential oil extraction was carried out with a 1 kg sample of *A. rugosa* for 1 h and 30 min using a steam distillation apparatus (EssenLab Plus, Hanil Lab Tech Co, Ltd., Yangju, Korea). The essential oil yield (%) was calculated as volume (mL) of extracted oil per 1 kg of fresh plant sample. After extraction, water and impurities in the extracted essential oil were removed using anhydrous sodium sulfate and stored at 4 °C for further analysis.

### 3.3. GC-MS Analysis 

The GC-MS analysis of the essential oil of *A. rugosa* was performed by a Varian CP-3800 (GC)/Varian 1200 L (MS) (Varian, Palo Alto, CA, USA) equipped with a capillary column VF-5MS (Agilent, Santa Clara, CA, USA) polydimethylsiloxane (30 m × 0.25 mm × 0.25 µm). The GC oven temperature was programmed to increase from 50 °C (for 5 min) to 250 °C (for 3 min) at a rate of 5 °C/min, then increased to 300 °C at a rate of 20 °C/min, and the final temperature was kept for 5 min. The injector temperature was set at 250 °C and the ion source temperature was set at 280 °C. One µL of the sample was injected with a split ratio of 20:1, and helium was used as a carrier gas at a rate of 1 mL/min. For mass spectra analysis, the ionization voltage was set to 70 eV, and the mass range was set to 30–500 *m*/*z*. The components in the essential oil of *A. rugosa* were identified by comparing the mass spectrum data of the National Institute of Standards and Technology (NIST, 3.0) library and the retention indices (RI) relative to a homologous series of n-alkanes (C_8_–C_20_) with those reported in the literature [34].

### 3.4. Statistical Analysis

Prior to the statistical analysis, the data of essential oil components obtained from 90 individuals of *A. rugosa* were integrated and sorted in ascending order based on the RI value. Then, the common chemical components that appeared in 40 or more *A. rugosa* populations were extracted separately from the raw data. Statistical analysis was done based on the completely extracted data and then cluster analysis was performed. Principal component analysis (PCA) was performed to analyze multiple data on the content of essential oil components and to understand the relationship between the essential oil composition and the collection site of the seeds of *A. rugosa*. All statistical analyzes were performed using IBM SPSS ver. 24 (IBM Corp. Released 2016, Chicago, IL, USA).

## 4. Conclusions

The results reveal a significant chemical polymorphism within Korean native *A. rugosa* populations according to seed collection sites. Based on the essential oil compositions, 90 individuals of *A. rugosa* were classified into six chemotypes: estragole, pulegone, methyl eugenol, menthone, isopulegone, and nepetalactone. Furthermore, these 90 individuals of *A. rugosa* could be classified into three groups—estragole, methyl eugenol, and pulegone—according to PCA and cluster analysis. These results can extend our knowledge of the chemical diversity of *A. rugosa* populations, suggesting the view that essential components are excellent markers at the intraspecific level. Further genetic diversity studies of *A. rugosa* populations are warranted to understand the variation within the species.

## Figures and Tables

**Table 1:** The extraction yield and color of essential oils from *A. rugosa* populations.

No.	Name (Sampling Site)	Yield (%)	Color	No.	Name (Sampling Site)	Yield (%)	Color
1	AR1(Incheon)	0.138	Colorless	46	AR46(NAC27)	0.140	Pale lemon
2	AR2(KNA1)	0.189	Lemon	47	AR47(NAC28)	0.164	Colorless
3	AR3(KNA2)	0.296	Colorless	48	AR48(NAC29)	0.269	Pale lemon
4	AR4(KNA3)	0.200	Colorless	49	AR49(NAC30)	0.170	Colorless
5	AR5(KNA4)	0.282	Colorless	50	AR50(NAC31)	0.225	Colorless
6	AR6(KNA5)	0.217	Colorless	51	AR51(Cheongju)	0.426	Colorless
7	AR7(KNA6)	0.261	Colorless	52	AR52(Seoul)	0.274	Colorless
8	AR8(KNA7)	0.282	Colorless	53	AR53(Chungnyeongsan)	0.170	Colorless
9	AR9(KNA8)	0.167	Colorless	54	AR54(Taebaek)	0.226	Pale lemon
10	AR10(KNA9)	0.151	Lemon	55	AR55(Hamyang)	0.863	Lemon
11	AR11(KNA10)	0.155	Colorless	56	AR56(Ulleungdo)	0.294	Colorless
12	AR12(KNA11)	0.217	Colorless	57	AR57(Dutasan)	0.227	Colorless
13	AR13(KU1)	0.121	Colorless	58	AR58(Bukhansan)	0.297	Pale lemon
14	AR14(KU2)	0.176	Lemon	59	AR59(Jeonju)	0.397	Colorless
15	AR15(KU3)	0.298	Colorless	60	AR60(Namyangju)	0.348	Colorless
16	AR16(KU4)	0.163	Colorless	61	AR61(Hwangmae-san)	0.371	Colorless
17	AR17(KU5)	0.153	Colorless	62	AR62(Jinju)	0.225	Colorless
18	AR18(KU6)	0.249	Colorless	63	AR63(Seoul)	0.298	Colorless
19	AR19(KU7)	0.261	Colorless	64	AR64(Seoul)	0.372	Pale lemon
20	AR20(KU8)	0.238	Colorless	65	AR65(Jirisan)	0.258	Pale lemon
21	AR21(NAC2)	0.259	Pale lemon	66	AR66(Chuncheon)	0.184	Colorless
22	AR22(NAC3)	0.287	Colorless	67	AR67(Seoul)	0.282	Lemon
23	AR23(NAC4)	0.108	Colorless	68	AR68(Gohan)	0.117	Pale lemon
24	AR24(NAC5)	0.247	Colorless	69	AR69(Hambaeksan)	0.295	Pale lemon
25	AR25(NAC6)	0.281	Colorless	70	AR70(Taebaek)	0.295	Pale lemon
26	AR26(NAC7)	0.216	Colorless	71	AR71(Seoul)	0.329	Pale lemon
27	AR27(NAC8)	0.250	Colorless	72	AR72(Yeongcheon)	0.322	Colorless
28	AR28(NAC9)	0.235	Colorless	73	AR73(Haman)	0.425	Colorless
29	AR29(NAC10)	0.247	Colorless	74	AR74(Gyeongju)	0.267	Colorless
30	AR30(NAC11)	0.259	Colorless	75	AR75(Gyeongju)	0.343	Colorless
31	AR31(NAC12)	0.290	Colorless	76	AR76(Jinju)	0.168	Colorless
32	AR32(NAC13)	0.200	Colorless	77	AR77(Hamyang)	0.262	Colorless
33	AR33(NAC14)	0.242	Pale lemon	78	AR78(Yeongcheon)	0.281	Colorless
34	AR34(NAC15)	0.239	Lemon	79	AR79(Pocheon)	0.179	Colorless
35	AR35(NAC16)	0.187	Pale lemon	80	AR80(Uiwang)	0.343	Colorless
36	AR36(NAC17)	0.160	Colorless	81	AR81(Pyeongnae)	0.278	Colorless
37	AR37(NAC18)	0.277	Lemon	82	AR82(Cheonmasan)	0.239	Pale lemon
38	AR38(NAC19)	0.240	Colorless	83	AR83(Odaesan)	0.457	Colorless
39	AR39(NAC20)	0.324	Colorless	84	AR84(Ulleungdo)	0.179	Lemon
40	AR40(NAC21)	0.273	Pale lemon	85	AR85Yeoncheon)	0.380	Pale lemon
41	AR41(NAC22)	0.383	Colorless	86	AR86(Choansan)	0.261	Pale lemon
42	AR42(NAC23)	0.338	Colorless	87	AR87(Changny-eong)	0.270	Colorless
43	AR43(NAC24)	0.255	Colorless	88	AR88(Balwangsan)	0.353	Colorless
44	AR44(NAC25)	0.440	Colorless	89	AR89(Jeongseon)	0.225	Pale lemon
45	AR45(NAC26)	0.181	Pale lemon	90	AR90(Seoul)	0.314	Colorless

KNA, Korea National Arboretum; KU, Korea University; NAC, National Agrobiodiversity Center.

**Table 2:** The chemotype classification of Korean *A. rugosa* populations.

Chemotype	Sample Name
Estragole (53)	AR3, AR5, AR8, AR10, AR11, AR12, AR15, AR16, AR18, AR19, AR20, AR22, AR23, AR24, AR25, AR26, AR27, AR28, AR29, AR30, AR31, AR32, AR33, AR34, AR51, AR52, AR55, AR56, AR58, AR59, AR60, AR61, AR62, AR63, AR64, AR67, AR68, AR71, AR72, AR73, AR74, AR75, AR76, AR77, AR78, AR79, AR80, AR81, AR82, AR85, AR86, AR87, AR90
Isopulegone (2)	AR83, AR88
Menthone (6)	AR6, AR9, AR17, AR35, AR36, AR42
Methyl eugenol (11)	AR4, AR13, AR45, AR46, AR47, AR48, AR49, AR50, AR53, AR84, AR89
Nepetalactone (1)	AR1
Pulegone (17)	AR2, AR7, AR14, AR21, AR37, AR38, AR39, AR40, AR41, AR43, AR44, AR54, AR57, AR65, AR66, AR69, AR70

**Table 3:** PC scores of the 32 components in the essential oils of Korean *A. rugosa* populations.

No.	Code	Compound Name	Principal Components			
			PC1	PC2	PC3	PC4
1	C1	1-Octen-3-ol	0.540	−0.130	0.029	0.296
2	C2	3-Octanone	0.463	0.046	0.005	0.228
3	C3	3-Octanol	0.586	−0.082	0.027	0.150
4	C4	β-Phellandrene	0.507	−0.157	0.164	0.331
5	C5	β-Pinene	0.745	−0.418	0.104	0.121
6	C6	D-Limonene	0.518	0.380	0.190	0.109
7	C7	Linalool	0.370	−0.236	0.043	−0.008
8	C8	1-Octen-3-yl-acetate	−0.273	0.181	0.021	0.086
9	C9	d-2,8-p-Menthadien-1-ol	0.500	−0.272	0.107	0.149
10	C10	Menthone	0.662	−0.444	0.195	0.551
11	C11	Isopulegone	0.186	−0.030	0.730	−0.625
12	C12	Estragole	−0.995	−0.077	−0.057	0.002
13	C13	Pulegone	0.753	−0.582	−0.269	−0.140
14	C14	Piperitone	0.405	−0.221	0.220	0.341
15	C15	Isopiperitenone	0.408	−0.201	0.529	−0.571
16	C16	α-Copaene	0.155	−0.098	0.078	0.222
17	C17	β-Bourbonene	0.472	0.242	0.190	0.263
18	C18	Methyl eugenol	0.347	0.919	−0.171	−0.001
19	C19	β-Elemene	0.265	0.142	0.081	0.198
20	C20	β-Caryophyllene	−0.029	0.706	−0.058	0.230
21	C21	α-Humulene	0.045	0.774	−0.037	0.239
22	C22	β-Cubebene	0.093	0.536	0.026	0.210
23	C23	γ-Elemene	0.071	0.306	0.148	0.265
24	C24	Butylated hydroxytoluene	0.128	0.146	0.025	−0.094
25	C25	γ-Cadinene	−0.188	0.046	0.237	−0.059
26	C26	β-Cadinene	0.126	0.152	0.189	0.200
27	C27	γ-Muurolene	−0.057	0.395	0.007	0.110
28	C28	β-Caryophyllene oxide	0.278	0.087	0.213	0.164
29	C29	T-Cadinol	0.426	0.060	0.189	0.147
30	C30	T-Muurolol	0.269	0.144	0.088	0.247
31	C31	α-Cadinol	0.448	0.157	0.111	0.259
32	C32	Phytol	0.088	0.441	−0.015	0.128
**% Variance**			**70.968**	**20.906**	**3.742**	**3.373**
**Cumulative % of variance**			**70.968**	**91.871**	**95.613**	**98.986**

**Table 4:** Chemical characterization of the principal components in essential oils of local varieties of *A. rugosa*.

Principal Component	Class	Corresponding Characters
PCA1	+	Pulegone, β-pinene, menthone
	−	Estragole
PCA2	+	Methyl eugenol, β-caryophyllene, α-humulene

**Table 5:** The different chemical characterization of the three groups for Korean *A. rugosa* populations.

Group	Major Chemical	Characterization
I	Estragole	Content ratio of estragole in essential oil is the highest
II	Methyl eugenol	The highest ratio of methyl eugenol content
III	Pulegone isopulegone	The highest ratio of pulegone and isopulegone contents
	Pulegone menthone	The highest ratio of pulegone and menthone contents (1:1)
	Pulegone	The highest ratio of pulegone content

## Data Availability

Not applicable.

## Supplementary Materials

The following supporting information can be downloaded at: https://www.mdpi.com/article/10.3390/molecules27196341/s1, Table S1. The area percent of 32 components in the essential oils of *A. rugosa* populations. Table S2. Correlation coefficients between 32 chemicals of essential oils from *A. rugosa* populations.
