# Supplementary material for: Chemical Diversity of Essential Oils from Korean Native Populations of Agastache rugosa (Korean Mint)
Source: Molecules. 2022 Sep 26;27(19):6341. doi: 10.3390/molecules27196341 (PMC9571081; doi:10.3390/molecules27196341)
Supplement: Supplementary file 1 [file molecules-27-06341-s001.zip › molecules-1929924-supplementary.pdf]

Table S1. The area percent of 32 components in the essential oils of *A. rugosa* populations.

RI*	Compound Name	Formula	Sample Name																									
			AR1	AR2	AR3	AR4	AR5	AR6	AR7	AR8	AR9	AR10	AR11	AR12	AR13	AR14	AR15	AR16	AR17	AR18	AR19	AR20	AR21	AR22	AR23	AR24	AR25	AR26
942	1-Octen-3-ol	$C_8H_{16}O$	0.15	1.74	0.77	1.09	1.00	1.24	1.81	1.28	2.07	0.61	1.20	0.88	2.04	4.20	0.78	1.00	1.44	0.39	1.09	0.71	1.75	0.78	0.83	0.78	0.97	0.84
944	3-Octanone	$C_8H_{16}O$	0.11	0.36	0.19	0.40	0.19	0.30	0.27	0.29	0.28	0.15	0.25	0.19	0.25	0.77	0.20	0.11	0.23	0.19	0.21	0.13	0.17	0.20	0.17	0.15	0.12	0.16
946	3-Octanol	$C_8H_{18}O$	0.00	0.07	0.00	0.04	0.02	0.03	0.04	0.00	0.00	0.01	0.00	0.01	0.06	0.12	0.02	0.01	0.04	0.01	0.01	0.01	0.02	0.02	0.01	0.01	0.00	0.01
971	β-Phellandrene	$C_{10}H_{16}$	0.03	0.46	0.02	0.05	0.01	0.19	0.10	0.00	0.12	0.02	0.00	0.01	0.05	0.21	0.03	0.03	0.12	0.00	0.01	0.01	0.15	0.00	0.00	0.00	0.00	0.01
989	β-Pinene	$C_{10}H_{16}$	0.19	0.80	0.05	0.16	0.03	0.89	1.11	0.07	0.85	0.07	0.04	0.03	0.35	1.87	0.07	0.02	0.82	0.04	0.04	0.04	0.87	0.02	0.03	0.01	0.00	0.03
1030	d-Limonene	$C_{10}H_{16}$	1.48	8.22	4.64	13.11	2.80	14.48	8.43	6.70	11.77	8.66	3.79	5.44	11.28	14.10	9.86	3.48	13.12	6.31	6.17	4.73	15.50	3.06	3.56	2.13	0.99	4.23
1092	Linalool	$C_{10}H_{18}O$	0.08	0.20	0.16	0.09	0.11	0.07	3.25	0.07	0.10	0.04	0.07	0.01	0.00	2.43	0.02	0.10	0.05	0.00	0.01	0.06	0.09	0.04	0.04	0.06	0.00	0.07
1108	1-Octen-3-yl-acetate	$C_{10}H_{18}O_2$	0.00	0.70	0.72	1.00	0.95	0.25	0.07	0.07	0.10	0.73	0.87	0.31	0.27	0.09	0.20	0.31	0.04	2.09	0.07	0.19	0.24	0.46	0.50	0.62	0.36	0.41
1122	d-2,8-p-Menthadien-1-ol	$C_{10}H_{16}O$	0.00	0.00	0.01	0.00	0.01	0.00	0.21	0.02	0.00	0.03	0.02	0.02	0.00	0.09	0.00	0.00	0.48	0.01	0.02	0.02	0.00	0.00	0.00	0.01	0.02	0.02
1154	Menthone	$C_{10}H_{18}O$	1.34	38.58	0.11	0.63	0.01	38.17	28.04	0.71	47.03	0.75	0.14	0.01	13.47	7.58	0.02	0.16	37.56	0.01	0.01	0.39	35.47	0.06	0.01	0.01	0.97	0.02
1172	Isopulegone	$C_{10}H_{16}O$	0.09	1.31	0.05	0.83	0.21	0.62	8.93	0.05	0.45	0.10	1.14	0.02	0.00	2.82	0.02	0.20	0.43	0.02	0.16	0.30	0.67	0.03	0.06	0.05	0.16	0.08
1192	Estragole	$C_{10}H_{12}O$	10.90	1.37	86.81	23.21	72.44	1.08	0.53	85.53	2.00	82.39	82.37	82.13	3.57	1.69	84.97	88.34	2.46	87.91	86.44	88.32	1.40	90.51	90.14	92.60	78.89	90.16
1227	Pulegone	$C_{10}H_{16}O$	2.90	31.37	0.17	0.89	0.16	30.21	39.00	0.85	22.19	1.10	0.60	0.02	13.63	51.87	0.00	0.31	23.87	0.04	0.03	0.74	33.32	0.03	0.05	0.01	1.04	0.03
1239	Piperitone	$C_{10}H_{16}O$	0.11	1.27	0.00	0.03	0.02	0.92	1.04	0.07	1.12	0.08	0.03	0.00	1.91	0.00	0.00	0.11	2.39	0.00	0.00	0.02	0.49	0.00	0.00	0.00	0.20	0.00
1254	Isopiperitenone	$C_{10}H_{14}O$	0.07	0.13	0.01	0.13	0.00	0.05	0.37	0.02	0.04	0.04	0.27	0.00	0.00	0.53	0.00	0.04	0.03	0.00	0.05	0.03	0.14	0.00	0.01	0.01	0.04	0.02
1358	α-Copaene	$C_{15}H_{24}$	0.00	0.02	0.02	0.02	0.03	0.01	0.01	0.01	0.02	0.01	0.00	0.00	0.00	0.00	0.00	0.00	0.03	0.00	0.01	0.00	0.00	0.01	0.01	0.00	0.05	0.01
1367	β-Bourbonene	$C_{15}H_{24}$	0.00	0.24	0.06	0.21	0.13	0.11	0.11	0.05	0.12	0.06	0.10	0.03	0.10	0.14	0.01	0.01	0.23	0.00	0.07	0.03	0.16	0.05	0.07	0.04	0.19	0.05
1381	Methyl eugenol	$C_{11}H_{14}O_2$	0.00	0.03	0.08	49.62	15.20	0.05	0.21	0.59	0.15	0.05	2.47	6.83	36.50	0.03	0.02	0.18	2.46	0.00	0.05	0.33	0.26	0.13	0.03	0.05	0.85	0.13
1384	β-Elemene	$C_{15}H_{24}$	0.00	0.17	0.10	0.13	0.15	0.11	0.08	0.05	0.13	0.09	0.08	0.03	0.10	0.17	0.00	0.01	0.22	0.01	0.08	0.04	0.11	0.07	0.06	0.06	0.25	0.06
1419	β-Caryophyllene	$C_{15}H_{24}$	1.14	3.91	3.09	3.50	2.57	2.70	0.42	1.52	4.28	2.07	2.30	1.99	5.42	0.42	2.31	3.68	3.51	1.48	2.95	2.06	2.48	2.12	2.09	1.52	6.40	1.57
1450	α-Humulene	$C_{15}H_{24}$	0.11	0.18	0.13	0.15	0.10	0.11	0.02	0.06	0.18	0.07	0.09	0.07	0.22	0.00	0.10	0.14	0.13	0.05	0.12	0.07	0.11	0.08	0.07	0.05	0.21	0.05
1478	β-Cubebene	$C_{15}H_{24}$	0.03	1.66	1.48	1.84	2.34	1.42	0.70	0.77	1.61	1.19	1.23	0.61	1.68	0.03	0.11	0.20	2.79	0.76	1.20	0.87	1.14	1.21	0.99	0.85	3.60	0.88
1494	γ-Elemene	$C_{15}H_{24}$	0.00	1.15	0.50	0.84	0.65	1.88	0.38	0.43	1.32	0.72	1.48	0.57	3.35	0.67	0.69	0.74	2.30	0.37	0.52	0.37	0.92	0.54	0.61	0.53	2.66	0.62
1500	Butylated hydroxytoluene	$C_{15}H_{24}O$	0.12	0.00	0.26	0.30	0.24	0.34	0.30	0.21	0.00	0.27	0.22	0.20	0.67	0.69	0.21	0.23	0.00	0.16	0.17	0.16	0.32	0.15	0.21	0.17	0.58	0.20
1513	γ-Cadinene	$C_{15}H_{24}$	0.00	0.03	0.01	0.02	0.01	0.04	0.02	0.00	0.04	0.01	0.02	0.01	0.08	0.02	0.11	0.02	0.07	0.05	0.01	0.00	0.03	0.11	0.00	0.01	0.06	0.01
1518	β-Cadinene	$C_{15}H_{24}$	0.00	0.16	0.05	0.09	0.00	0.00	0.06	0.00	0.17	0.07	0.00	0.00	0.41	0.14	0.06	0.08	0.24	0.04	0.05	0.00	0.15	0.06	0.06	0.00	0.27	0.00
1581	γ-Muurolene	$C_{15}H_{24}$	0.00	0.02	0.00	0.02	0.15	0.01	0.00	0.01	0.00	0.16	0.19	0.14	0.03	0.00	0.00	0.19	0.02	0.00	0.00	0.00	0.69	0.02	0.13	0.11	0.36	0.00
1588	β-Caryophyllene oxide	$C_{15}H_{24}O$	1.43	0.41	0.06	0.12	0.00	0.19	0.07	0.02	0.25	0.03	0.03	0.03	0.34	0.03	0.00	0.03	0.31	0.00	0.03	0.02	0.19	0.02	0.03	0.01	0.00	0.00
1646	t-Cadinol	$C_{15}H_{26}O$	0.00	0.05	0.02	0.02	0.01	0.08	0.04	0.00	0.05	0.01	0.00	0.00	0.12	0.09	0.00	0.00	0.00	0.00	0.01	0.00	0.04	0.00	0.00	0.00	0.04	0.00
1648	t-Muurolol	$C_{15}H_{26}O$	0.00	0.12	0.06	0.04	0.02	0.09	0.04	0.05	0.07	0.03	0.07	0.03	0.19	0.09	0.03	0.05	0.24	0.02	0.02	0.04	0.07	0.02	0.06	0.02	0.25	0.05
1659	α-Cadinol	$C_{15}H_{26}O$	0.00	0.20	0.00	0.08	0.05	0.22	0.08	0.00	0.22	0.07	0.00	0.00	0.56	0.22	0.04	0.06	0.50	0.00	0.04	0.00	0.26	0.03	0.00	0.00	0.00	0.00
2106	Phytol	$C_{20}H_{40}O$	0.00	0.04	0.02	0.01	0.01	0.04	0.00	0.00	0.07	0.03	0.04	0.00	0.19	0.01	0.01	0.04	0.02	0.00	0.04	0.02	0.00	0.00	0.00	0.00	0.08	0.00

* RI : retention time

(continued)

RI*	Compound Name	Formula	Sample Name																									
			AR27	AR28	AR29	AR30	AR31	AR32	AR33	AR34	AR35	AR36	AR37	AR38	AR39	AR40	AR41	AR42	AR43	AR44	AR45	AR46	AR47	AR48	AR49	AR50	AR51	AR52
942	1-Octen-3-ol	$C_8H_{16}O$	0.47	0.60	0.36	0.70	0.56	0.42	0.22	0.82	1.67	1.56	1.44	1.26	1.54	1.67	1.63	2.11	0.20	1.75	0.37	0.59	0.67	0.97	0.76	2.01	0.29	0.00
944	3-Octanone	$C_8H_{16}O$	0.10	0.13	0.08	0.13	0.09	0.11	0.07	0.13	0.27	0.21	0.29	0.21	0.24	0.24	0.25	0.36	0.00	0.27	0.20	0.11	0.55	0.21	0.13	0.34	0.03	0.14
946	3-Octanol	$C_8H_{18}O$	0.01	0.01	0.00	0.01	0.01	0.01	0.01	0.02	0.04	0.04	0.03	0.03	0.03	0.04	0.03	0.04	0.03	0.04	0.01	0.01	0.03	0.02	0.02	0.05	0.00	0.00
971	β-Phellandrene	$C_{10}H_{16}$	0.00	0.00	0.00	0.00	0.02	0.01	0.00	0.03	0.05	0.04	0.06	0.08	0.04	0.06	0.06	0.07	0.04	0.06	0.02	0.02	0.04	0.04	0.03	0.13	0.00	0.00
989	β-Pinene	$C_{10}H_{16}$	0.00	0.03	0.01	0.02	0.08	0.02	0.01	0.23	0.64	0.50	0.89	0.82	0.57	0.81	0.79	0.97	1.13	0.79	0.08	0.08	0.13	0.11	0.09	0.33	0.01	0.00
1030	d-Limonene	$C_{10}H_{16}$	0.76	4.41	1.99	2.43	11.12	2.47	1.29	5.19	5.35	4.36	5.84	5.96	5.26	6.67	6.26	7.43	0.00	5.24	10.73	14.38	17.22	16.13	11.04	34.66	1.64	0.65
1092	Linalool	$C_{10}H_{18}O$	0.06	0.03	0.03	0.02	0.09	0.04	0.04	0.08	0.32	0.30	0.50	0.51	0.56	0.52	0.87	1.38	0.28	0.30	0.04	0.11	0.06	0.10	0.07	0.09	0.00	0.01
1108	1-Octen-3-yl-acetate	$C_{10}H_{18}O_2$	1.06	0.58	0.23	0.59	0.83	0.59	0.18	0.10	0.02	0.04	0.02	0.03	0.03	0.02	0.04	0.04	0.03	0.04	0.10	0.09	0.09	0.33	0.20	0.99	0.11	0.38
1122	d-2,8-p-Menthadien-1-ol	$C_{10}H_{16}O$	0.00	0.01	0.00	0.00	0.02	0.03	0.01	0.12	0.19	0.12	0.00	0.15	0.20	0.00	0.14	0.20	0.31	0.15	0.03	0.06	0.01	0.02	0.03	0.05	0.00	0.00
1154	Menthone	$C_{10}H_{18}O$	0.01	0.00	0.05	0.01	0.10	0.11	0.06	9.65	57.53	50.94	28.13	22.68	24.68	17.22	21.53	42.19	27.05	26.74	0.12	0.06	0.04	0.20	0.16	0.08	0.01	0.44
1172	Isopulegone	$C_{10}H_{16}O$	0.10	0.05	0.05	0.02	0.31	0.05	0.03	17.01	0.75	0.60	0.91	1.04	1.00	1.07	1.13	0.72	0.96	1.07	0.39	0.25	0.18	0.22	0.56	0.06	0.01	0.02
1192	Estragole	$C_{10}H_{12}O$	94.50	88.21	92.01	91.17	79.25	89.79	92.09	44.25	2.57	2.21	0.86	1.50	0.33	0.56	1.41	1.66	0.14	0.24	2.23	1.19	2.20	1.48	4.25	1.95	94.96	91.31
1227	Pulegone	$C_{10}H_{16}O$	0.04	0.00	0.09	0.03	0.42	0.46	0.28	13.16	26.66	35.23	54.77	60.76	61.62	66.50	62.80	38.34	64.43	58.67	0.34	0.44	0.00	0.49	0.03	0.37	0.06	0.42
1239	Piperitone	$C_{10}H_{16}O$	0.00	0.00	0.00	0.00	0.01	0.04	0.03	0.17	1.16	0.73	1.01	0.46	0.21	0.00	0.18	0.00	0.51	0.00	0.03	0.02	0.00	0.01	0.00	0.01	0.00	0.02
1254	Isopiperitenone	$C_{10}H_{14}O$	0.01	0.00	0.00	0.00	0.11	0.03	0.03	0.16	0.08	0.11	0.15	0.10	0.00	0.13	0.22	0.29	0.14	0.21	0.04	0.04	0.05	0.04	0.00	0.03	0.00	0.00
1358	α-Copaene	$C_{15}H_{24}$	0.00	0.02	0.02	0.00	0.01	0.01	0.00	0.02	0.01	0.01	0.01	0.01	0.00	0.00	0.01	0.00	0.01	0.03	0.01	0.00	0.00	0.01	0.00	0.02	0.00	0.01
1367	β-Bourbonene	$C_{15}H_{24}$	0.06	0.06	0.05	0.05	0.07	0.09	0.04	0.11	0.00	0.16	0.11	0.08	0.00	0.00	0.07	0.12	0.10	0.09	0.18	0.11	0.15	0.11	0.12	0.23	0.00	0.00
1381	Methyl eugenol	$C_{11}H_{14}O_2$	0.08	0.06	0.06	0.41	0.10	1.12	0.95	0.00	0.02	0.03	0.02	0.15	0.26	0.05	0.02	0.02	0.41	0.29	73.78	71.88	69.77	69.07	73.12	51.83	0.74	0.22
1384	β-Elemene	$C_{15}H_{24}$	0.07	0.10	0.08	0.07	0.06	0.14	0.06	0.13	0.00	0.04	0.07	0.06	0.00	0.00	0.05	0.09	0.06	0.05	0.17	0.08	0.06	0.07	0.11	0.11	0.04	0.04
1419	β-Caryophyllene	$C_{15}H_{24}$	1.72	2.52	2.39	1.92	3.65	1.96	2.45	1.24	0.19	0.16	0.29	0.23	0.14	0.11	0.16	0.29	0.20	0.19	5.62	5.27	4.85	5.58	4.91	2.85	0.07	3.67
1450	α-Humulene	$C_{15}H_{24}$	0.05	0.09	0.08	0.06	0.13	0.07	0.09	0.05	0.00	0.00	0.01	0.00	0.00	0.00	0.00	0.00	0.01	0.00	0.26	0.23	0.22	0.23	0.23	0.13	0.06	0.12
1478	β-Cubebene	$C_{15}H_{24}$	0.28	1.55	1.42	0.92	0.95	0.94	0.84	1.77	0.35	0.28	0.58	0.57	0.25	0.00	0.29	0.80	0.49	0.49	2.46	2.03	1.44	1.54	1.30	1.92	0.93	1.05
1494	γ-Elemene	$C_{15}H_{24}$	0.13	0.78	0.46	0.78	0.96	0.67	0.45	1.96	0.11	0.05	0.23	0.18	0.06	0.01	0.06	0.33	0.21	0.14	1.12	1.30	0.99	1.30	0.99	0.59	0.62	0.61
1500	Butylated hydroxytoluene	$C_{15}H_{24}O$	0.16	0.22	0.19	0.17	0.16	0.20	0.17	0.37	0.17	0.16	0.24	0.18	0.16	0.01	0.10	0.31	0.25	0.20	0.31	0.28	0.22	0.26	0.24	0.24	0.12	0.21
1513	γ-Cadinene	$C_{15}H_{24}$	0.01	0.21	0.01	0.01	0.02	0.01	0.00	0.04	0.00	0.00	0.01	0.00	0.00	0.00	0.00	0.02	0.02	0.00	0.02	0.03	0.02	0.03	0.02	0.01	0.00	0.22
1518	β-Cadinene	$C_{15}H_{24}$	0.08	0.08	0.05	0.07	0.11	0.08	0.05	0.22	0.00	0.03	0.00	0.00	0.00	0.01	0.00	0.00	0.00	0.04	0.13	0.00	0.12	0.16	0.00	0.08	0.05	0.05
1581	γ-Muurolene	$C_{15}H_{24}$	0.01	0.00	0.14	0.15	0.26	0.14	0.02	0.41	0.15	0.00	0.00	0.00	0.09	0.00	0.03	0.00	0.00	0.00	0.35	0.38	0.24	0.37	0.00	0.05	0.19	0.00
1588	β-Caryophyllene oxide	$C_{15}H_{24}O$	0.00	0.03	0.00	0.02	0.04	0.03	0.06	0.06	0.02	0.03	0.00	0.00	0.00	0.00	0.00	0.00	0.00	0.00	0.12	0.09	0.11	0.11	0.20	0.02	0.00	0.02
1646	t-Cadinol	$C_{15}H_{26}O$	0.00	0.03	0.04	0.00	0.02	0.02	0.02	0.05	0.00	0.02	0.03	0.00	0.02	0.01	0.00	0.10	0.02	0.00	0.03	0.04	0.03	0.05	0.04	0.00	0.02	0.01
1648	t-Muurolol	$C_{15}H_{26}O$	0.04	0.05	0.02	0.10	0.04	0.00	0.07	0.09	0.08	0.00	0.04	0.06	0.04	0.04	0.05	0.02	0.04	0.06	0.06	0.07	0.05	0.07	0.05	0.04	0.04	0.06
1659	α-Cadinol	$C_{15}H_{26}O$	0.00	0.00	0.00	0.00	0.12	0.00	0.00	0.25	0.00	0.05	0.10	0.00	0.00	0.00	0.00	0.00	0.00	0.00	0.19	0.20	0.15	0.21	0.13	0.08	0.00	0.00
2106	Phytol	$C_{20}H_{40}O$	0.00	0.02	0.01	0.00	0.02	0.00	0.02	0.05	0.01	0.00	0.00	0.00	0.00	0.00	0.00	0.00	0.00	0.00	0.05	0.00	0.03	0.03	0.08	0.01	0.01	0.00

* RI : retention time

(continued)

RI*	Compound Name	Formula	Sample Name																									
			AR53	AR54	AR55	AR56	AR57	AR58	AR59	AR60	AR61	AR62	AR63	AR64	AR65	AR66	AR67	AR68	AR69	AR70	AR71	AR72	AR73	AR74	AR75	AR76	AR77	AR78
942	1-Octen-3-ol	$C_8H_{16}O$	0.65	0.70	0.38	0.28	1.00	0.29	0.47	0.49	0.59	0.40	0.83	0.47	0.78	0.60	0.44	0.57	0.64	0.64	0.44	0.54	0.44	0.45	0.45	0.25	0.88	0.59
944	3-Octanone	$C_8H_{16}O$	0.08	0.13	0.06	0.04	0.12	0.07	0.07	0.07	0.08	0.05	0.09	0.07	0.11	0.06	0.05	0.08	0.12	0.14	0.05	0.05	0.06	0.08	0.08	0.04	0.09	0.06
946	3-Octanol	$C_8H_{18}O$	0.00	0.02	0.01	0.00	0.02	0.00	0.00	0.00	0.00	0.01	0.02	0.00	0.00	0.00	0.00	0.00	0.02	0.01	0.00	0.00	0.00	0.01	0.00	0.00	0.00	0.00
971	β-Phellandrene	$C_{10}H_{16}$	0.00	0.04	0.00	0.00	0.07	0.00	0.01	0.00	0.00	0.00	0.00	0.00	0.06	0.00	0.00	0.00	0.00	0.03	0.00	0.00	0.00	0.00	0.00	0.00	0.00	0.00
989	β-Pinene	$C_{10}H_{16}$	0.02	0.43	0.04	0.01	0.57	0.01	0.02	0.01	0.02	0.01	0.18	0.02	0.56	0.33	0.02	0.03	0.38	0.44	0.01	0.01	0.02	0.00	0.02	0.01	0.01	0.01
1030	d-Limonene	$C_{10}H_{16}$	2.57	2.99	2.63	1.81	6.54	2.43	3.77	1.16	3.10	1.97	2.66	1.89	6.47	5.38	1.60	5.04	2.68	5.57	1.16	1.37	3.64	1.56	3.07	2.11	1.44	1.55
1092	Linalool	$C_{10}H_{18}O$	0.01	0.14	0.08	0.01	0.04	0.00	0.01	0.01	0.02	0.01	0.02	0.03	0.02	0.03	0.01	0.02	0.12	0.20	0.01	0.01	0.09	0.02	0.10	0.03	0.02	0.01
1108	1-Octen-3-yl-acetate	$C_{10}H_{18}O_2$	0.12	0.02	0.05	0.06	0.02	0.41	0.02	0.06	0.24	0.24	0.09	0.20	0.03	0.08	0.01	0.15	0.00	0.02	0.13	0.09	0.18	0.31	0.09	0.08	0.00	0.10
1122	d-2,8-p-Menthadien-1-ol	$C_{10}H_{16}O$	0.00	0.05	0.00	0.00	0.00	0.00	0.00	0.00	0.00	0.00	0.00	0.00	0.18	0.13	0.00	0.00	0.00	0.00	0.00	0.00	0.00	0.00	0.00	0.00	0.00	0.00
1154	Menthone	$C_{10}H_{18}O$	0.03	8.33	1.21	0.02	31.94	0.01	0.01	0.01	0.01	0.01	12.06	0.07	17.96	16.57	0.11	0.05	14.70	3.67	0.01	0.00	0.01	0.01	0.02	0.00	0.00	0.01
1172	Isopulegone	$C_{10}H_{16}O$	0.19	1.18	0.08	0.07	0.40	0.01	0.09	0.01	0.04	0.06	0.19	0.01	0.45	0.43	0.00	0.12	1.10	1.17	0.05	0.02	0.17	0.01	0.09	0.02	0.00	0.04
1192	Estragole	$C_{10}H_{12}O$	5.65	3.75	82.96	92.36	1.18	92.04	91.01	96.85	93.83	93.26	47.23	94.39	0.38	1.80	92.11	66.07	1.85	3.69	92.42	93.17	92.36	92.65	92.38	93.02	87.55	92.56
1227	Pulegone	$C_{10}H_{16}O$	0.11	73.72	4.18	0.11	47.22	0.07	0.07	0.00	0.04	0.03	26.37	0.17	64.91	67.06	0.58	0.44	73.77	79.42	0.05	0.03	0.03	0.05	0.05	0.05	0.07	0.03
1239	Piperitone	$C_{10}H_{16}O$	0.00	0.49	2.56	0.00	0.76	0.01	0.00	0.00	0.00	0.00	0.37	0.02	0.53	0.21	0.03	0.01	0.30	0.26	0.02	0.00	0.00	0.00	0.00	0.00	0.00	0.00
1254	Isopiperitenone	$C_{10}H_{14}O$	0.00	0.20	0.11	0.03	0.03	0.00	0.03	0.00	0.00	0.02	0.00	0.00	0.05	0.03	0.00	0.02	0.21	0.23	0.00	0.00	0.09	0.00	0.04	0.00	0.00	0.00
1358	α-Copaene	$C_{15}H_{24}$	0.00	0.01	0.00	0.00	0.02	0.01	0.01	0.00	0.00	0.00	0.01	0.00	0.00	0.01	0.00	0.01	0.01	0.00	0.00	0.00	0.00	0.00	0.00	0.00	0.00	0.00
1367	β-Bourbonene	$C_{15}H_{24}$	0.06	0.05	0.00	0.02	0.04	0.02	0.03	0.00	0.00	0.04	0.05	0.00	0.04	0.05	0.02	0.06	0.03	0.01	0.03	0.04	0.00	0.00	0.01	0.06	0.05	0.01
1381	Methyl eugenol	$C_{11}H_{14}O_2$	82.38	3.40	0.04	0.79	0.05	0.84	0.05	0.00	0.00	0.27	0.14	0.04	0.50	0.19	0.53	18.40	0.06	0.11	0.03	1.41	0.02	0.05	0.22	0.06	5.99	0.04
1384	β-Elemene	$C_{15}H_{24}$	0.07	0.06	0.00	0.05	0.12	0.05	0.06	0.00	0.02	0.05	0.10	0.01	0.08	0.10	0.03	0.09	0.06	0.02	0.07	0.05	0.00	0.04	0.03	0.00	0.05	0.04
1419	β-Caryophyllene	$C_{15}H_{24}$	4.03	0.22	1.72	1.84	2.21	1.58	2.08	0.92	0.00	1.83	3.55	1.21	1.13	3.39	2.05	3.08	0.16	0.31	2.97	1.46	2.24	3.06	2.21	2.10	1.02	2.77
1450	α-Humulene	$C_{15}H_{24}$	0.17	0.00	0.07	0.06	0.08	0.05	0.07	0.06	0.04	0.07	0.13	0.04	0.05	0.10	0.08	0.12	0.00	0.01	0.12	0.05	0.07	0.10	0.08	0.06	0.03	0.08
1478	β-Cubebene	$C_{15}H_{24}$	1.91	0.53	0.09	0.92	2.04	1.12	1.15	0.06	0.92	0.92	1.73	0.10	0.76	1.63	0.41	2.08	0.39	0.17	1.18	0.81	0.02	0.59	0.39	0.82	1.30	1.04
1494	γ-Elemene	$C_{15}H_{24}$	0.80	0.14	0.55	0.80	1.87	0.43	0.50	0.00	0.56	0.32	1.75	0.66	0.55	0.00	0.57	1.57	0.08	0.34	0.62	0.42	0.21	0.46	0.30	0.56	0.54	0.56
1500	Butylated hydroxytoluene	$C_{15}H_{24}O$	0.21	0.22	0.15	0.15	0.33	0.18	0.16	0.17	0.16	0.16	0.00	0.15	0.21	0.00	0.14	0.30	0.17	0.26	0.20	0.14	0.12	0.18	0.14	0.20	0.35	0.17
1513	γ-Cadinene	$C_{15}H_{24}$	0.02	0.00	0.01	0.01	0.04	0.10	0.01	0.00	0.01	0.10	0.03	0.01	0.02	0.00	0.00	0.03	0.00	0.01	0.01	0.11	0.00	0.00	0.01	0.17	0.01	0.13
1518	β-Cadinene	$C_{15}H_{24}$	0.00	0.04	0.07	0.05	0.21	0.03	0.04	0.02	0.00	0.03	0.16	0.01	0.00	0.00	0.04	0.15	0.03	0.00	0.05	0.00	0.02	0.04	0.03	0.00	0.04	0.01
1581	γ-Muurolene	$C_{15}H_{24}$	0.28	0.00	0.00	0.35	0.00	0.00	0.00	0.06	0.09	0.01	0.00	0.20	0.00	0.00	0.16	0.00	0.00	0.00	0.19	0.00	0.04	0.15	0.07	0.00	0.13	0.01
1588	β-Caryophyllene oxide	$C_{15}H_{24}O$	0.04	0.02	0.03	0.00	0.12	0.00	0.00	0.00	0.00	0.00	0.15	0.00	0.18	0.15	0.01	0.03	0.02	0.02	0.01	0.01	0.00	0.01	0.00	0.01	0.00	0.01
1646	t-Cadinol	$C_{15}H_{26}O$	0.02	0.02	0.01	0.01	0.08	0.00	0.00	0.01	0.00	0.00	0.03	0.00	0.05	0.00	0.00	0.03	0.00	0.01	0.00	0.00	0.00	0.00	0.00	0.00	0.03	0.01
1648	t-Muurolol	$C_{15}H_{26}O$	0.12	0.03	0.04	0.02	0.10	0.00	0.00	0.00	0.02	0.02	0.06	0.06	0.08	0.00	0.06	0.06	0.00	0.02	0.03	0.02	0.01	0.02	0.02	0.02	0.00	0.03
1659	α-Cadinol	$C_{15}H_{26}O$	0.00	0.08	0.12	0.04	0.24	0.00	0.00	0.00	0.00	0.00	0.15	0.00	0.23	0.00	0.00	0.17	0.03	0.09	0.05	0.00	0.02	0.00	0.00	0.00	0.00	0.00
2106	Phytol	$C_{20}H_{40}O$	0.17	0.00	0.06	0.00	0.01	0.06	0.03	0.00	0.01	0.00	0.00	0.00	0.00	0.00	0.04	0.15	0.02	0.00	0.02	0.03	0.01	0.01	0.00	0.00	0.00	0.00

* RI : retention time

(continued)

RI*	Compound Name	Formula	Sample Name											
			AR79	AR80	AR81	AR82	AR83	AR84	AR85	AR86	AR87	AR88	AR89	AR90
942	1-Octen-3-ol	$C_8H_{16}O$	0.28	0.45	0.39	0.00	0.00	0.57	0.56	0.34	0.38	0.58	1.51	0.55
944	3-Octanone	$C_8H_{16}O$	0.00	0.03	0.04	0.00	0.00	0.05	0.06	0.04	0.05	0.07	0.17	0.07
946	3-Octanol	$C_8H_{18}O$	0.00	0.00	0.00	0.00	0.00	0.00	0.00	0.00	0.00	0.02	0.03	0.01
971	β-Phellandrene	$C_{10}H_{16}$	0.00	0.00	0.00	0.00	0.00	0.00	0.00	0.00	0.00	0.00	0.00	0.00
989	β-Pinene	$C_{10}H_{16}$	0.00	0.00	0.01	0.00	0.54	0.02	0.14	0.02	0.01	0.25	0.04	0.01
1030	d-Limonene	$C_{10}H_{16}$	0.66	1.40	0.97	0.00	10.49	3.93	2.88	1.78	1.16	5.05	9.06	2.02
1092	Linalool	$C_{10}H_{18}O$	0.00	0.00	0.00	0.00	0.24	0.00	0.03	0.02	0.01	0.10	0.00	0.02
1108	1-Octen-3-yl-acetate	$C_{10}H_{18}O_2$	0.07	0.11	0.11	0.09	0.00	0.00	0.08	0.06	0.04	0.00	0.43	0.10
1122	d-2,8-p-Menthadien-1-ol	$C_{10}H_{16}O$	0.00	0.00	0.00	0.00	0.07	0.00	0.06	0.00	0.00	0.05	0.02	0.00
1154	Menthone	$C_{10}H_{18}O$	0.00	0.02	0.00	0.06	0.01	0.00	19.96	0.03	0.01	1.76	0.13	0.01
1172	Isopulegone	$C_{10}H_{16}O$	0.03	0.02	0.04	0.04	75.26	0.00	0.24	0.06	0.00	51.84	0.10	0.02
1192	Estragole	$C_{10}H_{12}O$	93.78	92.12	91.31	94.13	0.23	0.85	39.86	93.05	93.80	2.48	1.03	91.14
1227	Pulegone	$C_{10}H_{16}O$	0.00	0.13	0.87	0.15	6.07	0.03	28.01	0.15	0.06	32.58	0.14	0.05
1239	Piperitone	$C_{10}H_{16}O$	0.00	0.00	0.00	0.02	0.12	0.00	0.44	0.02	0.01	0.43	0.04	0.00
1254	Isopiperitenone	$C_{10}H_{14}O$	0.00	0.00	0.00	0.00	0.93	0.00	0.05	0.02	0.00	0.95	0.00	0.00
1358	α-Copaene	$C_{15}H_{24}$	0.00	0.00	0.00	0.00	0.00	0.00	0.00	0.01	0.00	0.00	0.00	0.01
1367	β-Bourbonene	$C_{15}H_{24}$	0.01	0.00	0.02	0.02	0.05	0.03	0.07	0.04	0.00	0.07	0.05	0.04
1381	Methyl eugenol	$C_{11}H_{14}O_2$	0.03	3.01	0.03	0.02	0.81	84.31	0.12	0.15	0.16	0.12	74.67	1.01
1384	β-Elemene	$C_{15}H_{24}$	0.04	0.00	0.06	0.05	0.03	0.06	0.08	0.08	0.00	0.05	0.06	0.07
1419	β-Caryophyllene	$C_{15}H_{24}$	2.82	1.10	3.45	3.00	0.19	5.37	2.68	1.54	1.35	0.12	6.42	2.01
1450	α-Humulene	$C_{15}H_{24}$	0.09	0.03	0.12	0.10	0.00	0.21	0.10	0.05	0.05	0.00	0.29	0.07
1478	β-Cubebene	$C_{15}H_{24}$	1.00	0.47	1.24	1.16	0.76	3.01	0.87	1.47	0.85	0.18	2.61	1.39
1494	γ-Elemene	$C_{15}H_{24}$	0.61	0.59	0.69	0.62	0.41	0.64	1.01	0.50	1.02	0.05	1.25	0.63
1500	Butylated hydroxytoluene	$C_{15}H_{24}O$	0.16	0.13	0.20	0.17	0.25	0.29	0.34	0.15	0.19	0.19	0.00	0.23
1513	γ-Cadinene	$C_{15}H_{24}$	0.01	0.21	0.01	0.01	0.16	0.01	0.00	0.01	0.02	0.00	0.02	0.01
1518	β-Cadinene	$C_{15}H_{24}$	0.01	0.04	0.08	0.06	0.06	0.00	0.10	0.06	0.09	0.00	0.13	0.06
1581	γ-Muurolene	$C_{15}H_{24}$	0.20	0.00	0.20	0.00	0.00	0.19	0.02	0.12	0.30	0.00	0.28	0.18
1588	β-Caryophyllene oxide	$C_{15}H_{24}O$	0.01	0.00	0.02	0.02	0.00	0.05	0.19	0.00	0.00	0.04	0.07	0.00
1646	t-Cadinol	$C_{15}H_{26}O$	0.04	0.00	0.00	0.03	0.05	0.00	0.02	0.00	0.03	0.00	0.02	0.00
1648	t-Muurolol	$C_{15}H_{26}O$	0.00	0.05	0.07	0.05	0.00	0.10	0.03	0.02	0.00	0.06	0.04	0.02
1659	α-Cadinol	$C_{15}H_{26}O$	0.00	0.00	0.00	0.00	0.00	0.00	0.13	0.04	0.07	0.00	0.12	0.04
2106	Phytol	$C_{20}H_{40}O$	0.00	0.00	0.00	0.00	0.00	0.11	0.00	0.06	0.00	0.00	0.00	0.03

* RI : retention time

Table S2. Correlation coefficients between 32 chemicals of essential oils from *A. rugosa* populations.

Traits	1)	2)	3)	4)	5)	6)	7)	8)	9)	10)	11)	12)	13)	14)	15)	16)
1)C1	1															
2)C2	**0.822** **	1														
3)C3	**0.826** **	**0.795** **	1													
4)C4	0.627 **	0.596**	0.691**	1												
5)C5	**0.743** **	0.602**	**0.745** **	0.679**	1											
6)C6	0.532**	0.611**	0.523**	0.491**	0.381**	1										
7)C7	0.608**	0.521**	0.580**	0.357**	0.682**	0.190	1									
8)C8	−0.007	0.169	0.051	0.055	−0.237*	0.193	−0.143	1								
9)C9	0.326**	0.214*	0.382**	0.235*	0.580**	0.181	0.397**	−0.212*	1							
10)C10	0.554**	0.367**	0.484**	0.582**	**0.750** **	0.220*	0.339**	−0.239*	0.569**	1						
11)C11	−0.096	−0.111	0.002	−0.034	00.133	0.096	0.093	−0.129	0.105	−0.035	1					
12)C12	−0.531**	−0.470**	−0.581**	−0.507**	−0.716**	−0.559**	−0.354**	0.253*	−0.479**	−0.630**	−0.219*	1				
13)C13	0.429**	0.280**	0.456**	0.375**	**0.756** **	0.098	0.401**	−0.332**	0.490**	0.635**	0.058	−0.687**	1			
14)C14	0.309**	0.177	0.358**	0.429**	0.432**	0.187	0.138	−0.169	0.416**	0.593**	0.027	−0.399**	0.325**	1		
15)C15	0.246*	0.212*	0.326**	0.188	0.473**	0.184	0.439**	−0.164	0.235*	0.159	**0.840** **	−0.419**	0.366**	0.113	1	
16)C16	0.218*	0.219*	0.183	0.254*	0.172	0.174	−0.001	0.177	0.254*	0.267*	−0.069	−0.153	0.119	0.213*	−0.048	1
17)C17	0.505**	0.566**	0.548**	0.593**	0.414**	0.633**	0.204	0.195	0.333**	0.364**	0.029	−0.500**	0.124	0.300**	0.177	0.559**
18)C18	0.047	0.177	0.108	−0.017	−0.152	0.470**	−0.104	0.053	−0.087	−0.199	−0.070	−0.403**	−0.223*	−0.102	−0.126	−0.059
19)C19	0.368**	0.362**	0.344**	0.425**	0.274**	0.347**	0.118	0.173	0.233*	0.213*	−0.047	−0.282**	0.062	0.238*	0.037	0.672**
20)C20	−0.065	0.016	−0.097	0.024	−0.345**	0.282**	−0.325**	0.188	−0.244*	−0.232*	−0.228*	−0.025	−0.453**	0.012	−0.374**	0.189
21)C21	−0.047	0.060	−0.063	0.079	−0.315**	0.351**	−0.314**	0.168	−0.246*	−0.214*	−0.221*	−0.107	−0.448**	0.028	−0.360**	0.137
22)C22	0.013	0.019	−0.049	0.068	−0.175	0.259*	−0.236**	0.167	0.009	−0.052	−0.104	−0.134	−0.276**	0.064	−0.260*	0.535**
23)C23	0.173	0.121	0.132	0.190	−0.023	0.282**	−0.169	0.079	−0.005	0.064	−0.078	−0.106	−0.207	0.356**	−0.171	0.337**
24)C24	0.323**	0.317**	0.319**	0.073	0.211*	0.215*	0.293**	0.005	−0.033	−0.049	0.069	−0.143	0.012	0.020	0.198	0.120
25)C25	−0.169	−0.118	−0.106	−0.061	−0.111	−0.027	−0.102	0.066	−0.076	−0.127	0.181	0.171	−0.221*	−0.014	0.009	0.051
26)C26	0.281**	0.207	0.307**	0.266*	0.107	0.299**	−0.030	0.081	0.035	0.131	−0.006	−0.153	−0.080	0.409**	−0.047	0.390**
27)C27	−0.063	−0.097	−0.156	−0.068	−0.207*	0.126	−0.177	0.023	−0.141	−0.143	−0.074	0.028	−0.287 **	−0.185	−0.152	−0.011
28)C28	0.048	0.105	0.092	0.296**	0.130	0.132	−0.039	−0.066	0.047	0.169	−0.042	−0.321**	0.033	0.259*	−0.023	0.023
29)C29	0.445**	0.373**	0.445**	0.431**	0.471**	0.346**	0.327**	−0.128	0.091	0.362**	0.112	−0.440**	0.212*	0.301**	0.207	0.103
30)C30	0.339**	0.250*	0.302**	0.344**	0.233*	0.230*	0.012	0.002	0.297**	0.257*	−0.055	−0.285 **	0.057	0.471**	−0.027	0.427**
31)C31	0.409**	0.338**	0.440**	0.461**	0.361**	0.526**	0.053	−0.072	0.262*	0.356**	−0.044	−0.469 **	0.172	0.625**	0.006	0.185
32)C32	0.067	0.002	0.006	0.034	−0.115	0.095	−0.143	−0.037	−0.160	−0.061	−0.077	−0.120	−0.204	0.234*	−0.142	0.108

Traits	17)	18)	19)	20)	21)	22)	23)	24)	25)	26)	27)	28)	29)	30)	31)	32)
17)C17	1															
18)C18	0.341**	1														
19)C19	**0.793** **	0.195	1													
20)C20	0.337**	0.640**	0.448**	1												
21)C21	0.361**	**0.718** **	0.415**	**0.965** **	1											
22)C22	0.511**	0.521**	**0.711** **	**0.729** **	**0.701** **	1										
23)C23	0.424**	0.268*	0.594**	0.623**	0.598**	0.667**	1									
24)C24	0.291**	0.162	0.358**	0.126	0.094	0.189	0.418**	1								
25)C25	−0.047	−0.061	−0.023	0.114	0.054	0.110	0.184	0.059	1							
26)C26	0.442**	0.133	0.572**	0.490**	0.465**	0.495**	**0.808** **	0.391**	0.144	1						
27)C27	0.150	0.347**	00.168	0.385**	0.400**	0.348**	0.317**	0.181	−0.179	0.250*	1					
28)C28	0.163	0.081	00.127	0.165	0.284**	0.055	0.172	−0.048	−0.051	0.192	−0.060	1				
29)C29	0.383**	0.164	0.437**	0.204	0.221*	0.252*	0.544**	0.576**	0.073	0.522**	0.074	0.166	1			
30)C30	0.470**	0.205	0.592**	0.439**	0.400**	0.519**	**0.720** **	0.346**	0.086	0.627**	0.189	0.151	0.346**	1		
31)C31	0.512**	0.261*	0.515**	0.383**	0.421**	0.378**	**0.733** **	0.321**	0.039	**0.734** **	0.160	0.323**	0.608**	0.572**	1	
32)C32	0.171	0.442**	0.281**	0.478**	0.482**	0.449**	0.538**	0.349**	0.036	0.433**	0.113	0.107	0.300**	0.468**	0.386**	1

1)C1: 1-Octen-3-ol, 2)C2: 3-Octanone , 3)C3: 3-Octanol, 4)C4: β-Phellandrene, 5)C5: l-β-Pinene, 6)C6: d-Limonene, 7)C7: Linalool, 8)C8: 1-Octen-3-yl-acetate, 9)C9: d-2,8-p-menthadien-1-ol, 10)C10: Menthone, 11)C11:Isopulegone, 12)C12: Estragole, 13)C13: Pulegone, 14)C14: Piperitone, 15)C15: (S)-Isopiperitenone, 16)C16: α-Copaene, 17)C17: β-Bourbonene, 18)C18: Methyl eugenol, 19)C19: (-)-β-Elemene, 20)C20: β-Caryophyllene, 21)C21: α-Humulene, 22)C22: β-Cubebene, 23)C23: γ-Elemene, 24)C24: Butylated hydroxytoluene, 25)C25: (R)-γ-Cadinene, 26)C26: β-Cadinene, 27)C27: γ-Muurolene, 28)C28: β-Caryophyllene oxide, 29)C29: t-Cadinol, 30)C30: t-Muurolol, 31)C31: α-Cadinol, 32)C32: Phytol.

* Significant at 5% level of probability ($P < 0.05$).

** Significant at 1% level of probability ($P < 0.01$).
